# EASIX and m-EASIX predict severe cytokine release syndrome and overall survival after CAR T-cell therapy

**DOI:** 10.1016/j.bvth.2024.100025

**Published:** 2024-08-21

**Authors:** Eleni Gavriilaki, Ifigeneia Tzannou, Ioannis Batsis, Ioannis Tsonis, Maria Liga, Konstantinos Gkirkas, Maria Ximeri, Panagiotis Dolgyras, Vasiliki Bampali, Paschalis Evangelidis, Zoi Bousiou, Anna Vardi, Christos Demosthenous, Eulampia Stroggyli, Maria Bouzani, Eleftheria Sagiadinou, Despina Mallouri, Tatiana Tzenou, Damianos Sotiropoulos, Stavros Gigantes, Achilles Anagnostopoulos, Dimitrios Karakasis, Helen Papadaki, Panagiotis Tsirigotis, Alexandros Spyridonidis, Theodoros Vassilakopoulos, Maria Angelopoulou, Ioanna Sakellari, Ioannis Baltadakis

**Affiliations:** 1 Hematology Department, Bone Marrow Transplantation Unit, G. Papanicolaou General Hospital of Thessaloniki, Thessaloniki, Greece; 2 Second Propedeutic Department of Internal Medicine, Hematology Unit, Hippocration Hospital, Aristotle University of Thessaloniki, Thessaloniki, Greece; 3 Hematology and Lymphomas Department and Bone Marrow Transplantation Unit, Evangelismos General Hospital, Athens, Greece; 4 Hematology Department, Bone Marrow Transplantation Unit and Institute of Cell Therapy, University of Patras, Rio, Greece; 5 Second Department of Internal Medicine, Hematology Unit, National and Kapodistrian University of Athens, School of Medicine, Attikon University Hospital, Athens, Greece; 6 Department of Hematology, University Hospital of Heraklion, Heraklion, Greece; 7 Department of Haematology and Bone Marrow Transplantation, School of Medicine, Laikon General Hospital, National and Kapodistrian University of Athens, Athens, Greece

**TO THE EDITOR:**

Immunotherapy with chimeric antigen receptor T (CAR T) cells has revolutionized the management of patients with relapsed/refractory B-lymphoproliferative neoplasms, with notably effective clinical responses. Many challenges limit the therapeutic efficacy of CAR T-cell therapies including infectious complications, endothelial injury syndromes, and neurotoxicity.^1^^,^^2^ Cytokine release syndrome (CRS) and immune effector cell–associated neurotoxicity syndrome (ICANS) are major complications affecting patients who receive CAR T-cell therapies, leading to increased morbidity and mortality.^3^ Endothelial dysfunction and activation play a substantial role in the pathogenesis of these syndromes.^4^

The role of endothelial dysfunction in the pathophysiology of acute graft-versus-host-disease and in other complications of allogeneic hematopoietic cell transplantation has been supported.^5^ Thus, endothelial activation and stress index (EASIX; lactate dehydrogenase [LDH] [units per liter] × creatinine [milligrams per deciliter]/platelets [PLTs] [× 10^9^ per liter]) has been used as a marker of endothelial damage and as a predictor of outcomes and survival in patients who undergo allogeneic hematopoietic cell transplantation.^6^^,^^7^ Recently, the modified EASIX (m-EASIX), in which creatinine is replaced by C-reactive protein (CRP; milligrams per deciliter), was introduced.^8^ In this multicenter study, we examined whether EASIX and m-EASIX, as markers of endothelial injury, can predict the onset of CRS and ICANS in patients who receive CAR T-cell therapy. Furthermore, we tested the hypothesis that EASIX and m-EASIX are potential markers of the severity of CAR T cell–related toxicity and survival in those patients.

In this real-world multicenter retrospective study, consecutive adult patients (aged ≥18 years) who received CAR T-cell therapy for relapsed/refractory B-cell acute lymphoblastic leukemia or lymphomas were included. Patients were treated with commercially available CAR T-cell products according to current indications at 6 centers in Greece between 2020 and 2023. Before CAR T-cell infusion, all patients received lymphodepleting chemotherapy (LDC) with cyclophosphamide and fludarabine.^9^ Extraction of clinical and laboratory data from patients’ medical records was performed. The severity of CRS and ICANS was evaluated according to the American Society for Transplantation and Cellular Therapy consensus grading system.^10^ Grade 3 or 4 CRS and ICANS were considered severe. Patient laboratory data (ferritin, LDH, PLTs, creatinine, and CRP) were recorded before the administration of LDC (baseline), at day 0 (CAR T-cell therapy infusion), and day 14. EASIX was calculated by the formula LDH (units per liter) × creatinine (milligrams per deciliter)/PLTs (× 10^9^ cells per liter) and m-EASIX by the formula LDH (units per liter) × CRP (milligrams per deciliter)/PLTs (× 10^9^ cells per liter) for every participant of the study at specific time points (baseline, day 0, and day 14).^6^ The best overall response was evaluated between month 1 and month 12 after the administration of CAR T-cell therapy. The minimum follow-up time for inclusion in the study was 1 month.

This study was conducted according to the Declaration of Helsinki and was approved by the G. Papanicolaou General Hospital of Thessaloniki Institutional Review Board (protocol 753/2019). All participants provided written informed consent to participate in the current study.

For the statistical analysis, SPSS 22.0 (IBM SPSS Statistics for Windows, version 22.0; IBM Corp, Armonk, NY) was used. Statistical significance was set at *P* < .05. Results are presented as median and range for nonnormal distribution variables and as frequency for categorical variables. The data were analyzed on 30 June 2023. In all EASIX/m-EASIX scores, a binary logarithm transformation (log_2_) was applied for the primary statistical analysis. For survival analysis, the Kaplan-Meier method was used, and the survival curves of the different groups were compared with the log-rank test. Receiver operating characteristic (ROC) curves were built for analysis of specificity, and sensitivity.

**Figure 1.**
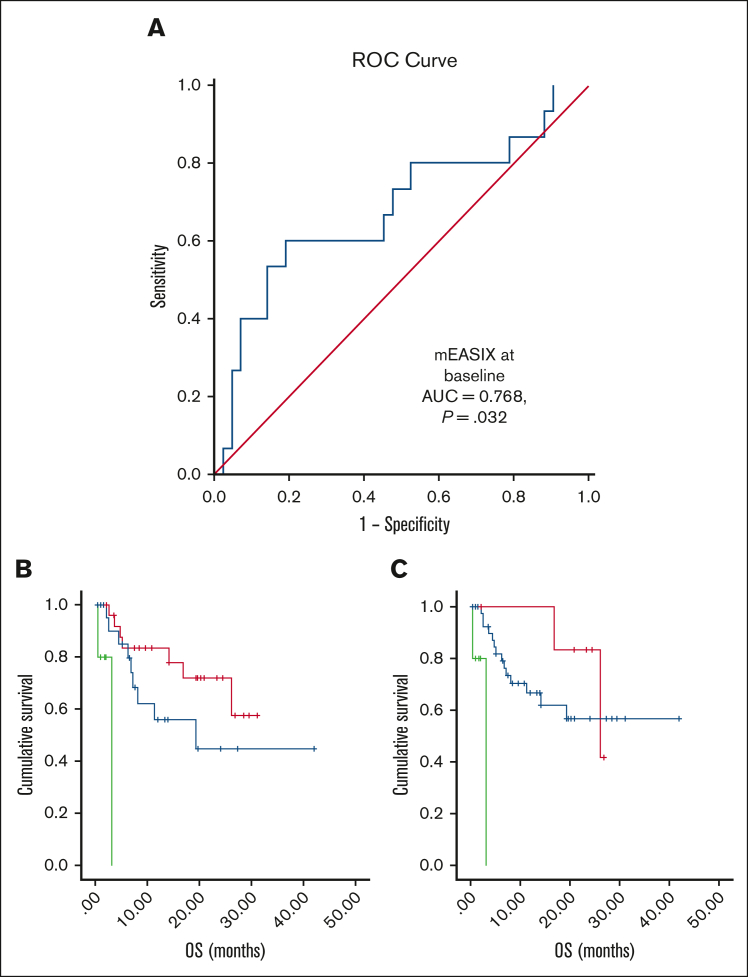
**Prediction of CRS by mEASIX at baseline and Kaplan-Meier curves of OS.** (A) ROC curve for prediction of severe CRS by m-EASIX: onset of severe CRS was predicted by baseline m-EASIX (AUC = 0.768; *P* = .032). 1 − Specificity stands for the probability that a true negative will test positive. (B,C) Kaplan-Meier curves of the OS rate in our study population: MCL disease (*P* < .001) and infusion of brexucabtagene autoleucel (*P* < .001) associated with a poor OS in comparison with the other diagnoses and products; (B) red, axicabtagene ciloleucel; blue, tisagenlecleucel; and green, brexucabtagene autoleucel; (C) red, B-ALL; blue, DLBCL, TFL, and PMBCL; and green, MCL. AUC, area under the curve; B-ALL, B-cell acute lymphoblastic leukemia; DLBCL, diffuse large B-cell lymphoma; MCL, mantle cell lymphoma; PMBCL, primary mediastinal large B-cell lymphoma; TFL, transformed follicular lymphoma.

A total of 90 patients were included. In Table 1, the demographic and clinical characteristics of the patients are summarized. CRS of grade ≥3 was diagnosed at a median of 5 days (range, 1-6 days). CRS was correlated with m-EASIX (*P* = .025) and serum levels of CRP (*P* = .001) at baseline. m-EASIX at baseline predicted the onset of severe CRS in ROC analysis (area under the curve = 0.768; *P* = .032), as shown in Figure 1. By contrast, severe ICANS was not associated with EASIX markers. However, increased serum levels of LDH at baseline were correlated with the onset of severe ICANS (*P* = .001).

**Table 1.** **Patient characteristics**

Patient characteristics	Total (N = 90)
Age at CAR T-cell infusion, median (range), y	49 (18-75)
**Diagnosis, n (%)**	
B-ALL	10 (11%)
NHL	80 (89%)
DLBCL	56 (62%)
PMBCL	11 (12%)
TFL	8 (9%)
MCL	5 (6%)
**CAR T-cell product, n (%)**	
Axicabtagene ciloleucel	42 (47%)
Tisagenlecleucel	37 (41%)
Brexucabtagene autoleucel	11 (12%)
Prior lines of treatment, median (range)	3 (1-9)
Previous autologous transplantation	13 (15%)
Previous allogeneic transplantation	7 (8%)
**CRS or ICANS**	
CRS grade ≥3	19 (21%)
ICANS grade ≥3	32 (34%)
**Laboratory findings at baseline (range)**	
LDH, IU/L	248 (125-1411)
Creatinine, mg/dL	0.8 (0.39-7.0)
PLTs, × 10^9^ cells per L	77.5 (16.0-314)
CRP, mg/dL	6.1 (0.28-86.3)

B-ALL, B-cell acute lymphoblastic leukemia; DLBCL, diffuse large B-cell lymphoma; MCL, mantle cell lymphoma; NHL, non-Hodgkin lymphoma; PMBCL, primary mediastinal large B-cell lymphoma; TFL, transformed follicular lymphoma.

With a median follow-up of 6.6 months (range, 1-42 months), progression-free survival (PFS) and overall survival (OS) rates at 1 year were 33.2% and 62.5%, respectively. Mantle cell lymphoma diagnosis in combination with the infusion of brexucabtagene autoleucel, was associated with poor OS in comparison with other diagnoses/products (both *P* < .001; Figure 1). Serum levels of ferritin and LDH at baseline, CRP levels at every time point, EASIX14, and m-EASIX14 were correlated with OS. EASIX14 and m-EASIX14 predicted the risk of death in the ROC curves. Likewise, EASIX14 was associated with PFS.

Our real-world data showed that among the 90 patients included in our analysis, 21% and 39% developed severe (grade ≥3) CRS and ICANS, respectively, results similar to reported data from published studies.^8^^,^^11^^,^^12^ Early recognition of CRS and ICANS and initiation of tocilizumab and corticosteroids upon indication are crucial for the management and prevention of these complications.^13^ Our study is among the first in which the onset of severe CRS was correlated with baseline m-EASIX and CRP. In the study of Acosta-Medina et al, the EASIX score at any time point did not predict the onset of severe CRS or ICANS.^14^ However, in the study of Korell et al, EASIX calculated before the administration of LDC was associated with severe CRS.^12^ Similarly, Greenbaum et al in their study correlated the baseline EASIX score combined with ferritin levels (before the administration of LDC) with CRS onset in patients treated with axicabtagene ciloleucel.^11^ Moreover, they showed that baseline EASIX in combination with CRP and ferritin levels could predict ICANS development.^11^ Pennisi et al were the first to report that m-EASIX (calculated at baseline and early after CAR T-cell infusion) was associated with the development of severe CRS and ICANS.^8^ In addition to their findings, in our study, EASIX and m-EASIX scores (at day 14) were correlated with OS. The EASIX score has been shown as a reliable predictor of bleeding events after CAR T-cell treatment.^15^

In our study, elevated levels of LDH at baseline were correlated with severe ICANS (*P* = .001), in agreement with findings from previous studies.^11^^,^^16^ As mentioned above, we failed to show a correlation between EASIX/m-EASIX scores and ICANS development. However, in the study of de Boer et al, the EASIX score calculated before LDC was associated with ICANS grade ≥2 onset.^17^ Anakinra (an interleukin 1 receptor antagonist) and defibrotide have been used as prophylactic agents for ICANS in phase 1 and phase 2 clinical trials.^18^

In this real-world study, ferritin and LDH levels at baseline, CRP levels at every time point, EASIX14, and m-EASIX14 were associated with OS. As shown before, elevated CRP, ferritin, LDH, and interleukin-6 serum levels before the administration of CAR T-cell therapy are correlated with poor OS.^19^^,^^20^ To our knowledge, this is the first study to correlate EASIX and m-EASIX scores with OS of patients who receive CAR T-cell therapy. Moreover, EASIX14 was associated with PFS. Likewise, in the study of Vercellino et al, increased LDH levels at baseline in addition to other clinical and laboratory factors predicted poor PFS.^19^

In conclusion, in our retrospective study, m-EASIX before LDC administration was predictive for the development of severe CRS after CAR T-cell infusion. Thus, this score can be helpful for the identification of patients who are at increased risk for CRS before the onset of the symptoms. Moreover, we showed that EASIX14 and m-EASIX14, calculated early after infusion, were associated with OS. These findings can be implemented in clinical practice for the recognition of patients who are at greater risk of death.

**Conflict-of-interest disclosure:** The authors declare no competing financial interests.
